# Framing rehabilitation through health policy and systems research: priorities for strengthening rehabilitation

**DOI:** 10.1186/s12961-022-00903-5

**Published:** 2022-09-20

**Authors:** Alarcos Cieza, Aku Kwamie, Qhayiya Magaqa, Nino Paichadze, Carla Sabariego, Karl Blanchet, Nukhba Zia, Abdulgafoor M. Bachani, Abdul Ghaffar, Bente Mikkelsen

**Affiliations:** 1 Sensory Functions, Disability and Rehabilitation Unit, Department of Noncommunicable Diseases, World Health Organization, Avenue Appia 20, 1211 Geneva 27, Switzerland; 2 Alliance for Health Policy and Systems Research, World Health Organization, Avenue Appia 20, 1211 Geneva 27, Switzerland; 3 Center on Commercial Determinants of Health, Milken Institute School of Public Health, The George Washington University, 950 New Hampshire Avenue, NW Washington DC, 20052 United States of America; 4 Department of Health Sciences and Medicine, University of Lucerne, Frohburgstrasse 3, P.O. Box 4466, 6002 Lucerne, Switzerland; 5 Geneva Centre of Humanitarian Studies, Université de Genève, The Graduate Institute (IHEID), 28, Boulevard du Pont-d’Arve, 1205 Geneva, Switzerland; 6 International Health, Health Systems Division, International Injury Research Unit, Johns Hopkins Bloomberg School of Public Health, 615 N. Wolfe Street, Baltimore, MD 21205 United States of America; 7 Department of Noncommunicable Diseases, World Health Organization, Avenue Appia 20, 1211 Geneva 27, Switzerland

**Keywords:** Rehabilitation, Health policy, Health systems strengthening, Systems thinking

## Abstract

**Background:**

Recent estimates report that 2.4 billion people with health conditions globally could benefit from rehabilitation. While the benefits of rehabilitation for individuals and society have been described in the literature, many individuals, especially in low- and middle-income countries do not have access to quality rehabilitation. As the need for rehabilitation continues to increase, it is crucial that health systems are adequately prepared to meet this need. Practice- and policy-relevant evidence plays an important role in health systems strengthening efforts. The aim of this paper is to report on the outcome of a global consultative process to advance the development of a research framework to stimulate health policy and systems research (HPSR) for rehabilitation, in order to generate evidence needed by key stakeholders.

**Methods:**

A multi-stakeholder participatory technical consultation was convened by WHO to develop a research framework. This meeting included participants from selected Member States, rehabilitation experts, HPSR experts, public health researchers, civil society and other stakeholders from around the world. The meeting focused on introducing systems approaches to stakeholders and deliberating on priority rehabilitation issues in health systems. Participants were allocated to one of four multi-stakeholder groups with a facilitator to guide the structured technical consultations. Qualitative data in the form of written responses to guiding questions were collected during the structured technical consultations. A technical working group was then established to analyse the data and extract its emerging themes. This informed the development of the HPSR framework for rehabilitation and a selection of preliminary research questions that exemplify how the framework might be used.

**Results:**

A total of 123 individuals participated in the multi-stakeholder technical consultations. The elaborated framework is informed by an ecological model and puts forth elements of the six WHO traditional building blocks of the health system, while emphasizing additional components pertinent to rehabilitation, such as political priority, engagement and participatory approaches, and considerations regarding demand and access. Importantly, the framework highlights the multilevel interactions needed across health systems in order to strengthen rehabilitation. Additionally, an initial set of research questions was proposed as a primer for how the framework might be used.

**Conclusions:**

Strengthening health systems to meet the increasing need for rehabilitation will require undertaking more HPSR to inform the integration of rehabilitation into health systems globally. We anticipate that the proposed framework and the emerging research questions will support countries in their quest to increase access to rehabilitation for their populations.

## Background

Rehabilitation refers to “a set of interventions designed to optimize functioning and reduce disability in individuals with health conditions in interaction with their environment” [1], where “health condition” refers to disease (acute or chronic), disorder, injury or trauma. Rehabilitation is relevant because it addresses functioning, which has been advanced as the third public health indicator, alongside mortality and morbidity [2].

The effects of a lack of access to appropriate rehabilitation services can be seen in worsened individual health outcomes and limited participation in society; this in turn has a marked effect on the achievement of several Sustainable Development Goals (SDGs), including SDG 3 (good health and well-being) and SDG 8 (decent work and economic growth) [3]. Recent evidence has also shown significant economic benefits to governments, with marked returns on investment when rehabilitation, including assistive technology, is provided to individuals [4, 5].

Despite the benefits of rehabilitation to individuals and society at large, there remain persisting needs for rehabilitation globally. According to recent estimates, 2.4 billion people with health conditions could be in need of rehabilitation globally; this need has increased by 63% since 1990 [6] and is expected to continue to rise [7, 8]. This is owing to the increase in aging populations, chronic health conditions and injuries [1].

Significantly, however, this need for rehabilitation is not matched with the demand for rehabilitation. Here “demand” refers to the number of people actually accessing services and therefore represents a proportion of the total need. The provision of rehabilitation has generally been under-prioritized in countries. Limitations posed by human and financial resource constraints to provide rehabilitation are factors [9]. Rehabilitation has also historically been seen as a specialized service for people with disabilities and not as an essential service that can be beneficial to all in need [1, 10]. As a result, rehabilitation has not been seen as a political priority within health systems. Recognizing the gaps in the availability and accessibility of rehabilitation globally, the Rehabilitation 2030 initiative was launched in 2017 [11]. A key area of action of the Rehabilitation 2030 initiative commits stakeholders to building research capacity and expanding the availability of robust evidence for rehabilitation.

Multidisciplinary in nature, health policy and systems research (HPSR) aims to draw a comprehensive picture of how health systems respond and adapt to health policies. HPSR also aims to describe how health policies can shape and are themselves shaped by health systems and the broader determinants of health [12]. Such a perspective lends itself to the emerging questions for rehabilitation in the twenty-first century, the interventions for which are multilevel and need to be embedded within health systems. Therefore, rehabilitation can benefit from HPSR approaches, such as systems thinking [13, 14] and embedded implementation research [15, 16]. HPSR brings together political actors, practitioners and researchers to understand the problems and identify solutions by generating new knowledge and evidence. For this reason, HPSR is useful to make rehabilitation a political priority.

Situating rehabilitation within health systems instead of as a vertical programme driven by stand-alone delivery mechanisms enables a more holistic appreciation of what comprises rehabilitation, while recognizing that rehabilitation is susceptible to broader systemic influences. To date, however, there has been little guidance on how HPSR might be applied to rehabilitation. In the context of achieving universal health coverage (UHC) and the SDGs, an HPSR approach towards rehabilitation is needed. Given these gaps, this paper presents a framework for stimulating HPSR for the purpose of strengthening rehabilitation and proposes an initial set of research questions to demonstrate the potential of the framework.

## Methods

### Design

In July 2019, the Rehabilitation Programme and the Alliance for Health Policy and Systems Research (the Alliance) at WHO jointly organized a 2-day global consultative meeting in Geneva. The aim of the meeting was to develop an emerging HPSR agenda for rehabilitation. Participants from selected Member States, rehabilitation experts, HPSR experts, public health researchers, civil society and other stakeholders were invited [17]. Specifically, the objectives of the meeting were to sensitize stakeholders to systems thinking (that is, an approach to problem-solving that views problems as part of a wider dynamic system and prioritizes the understanding of linkages, relationships, interactions and interdependencies among the components of a system that gives rise to the system’s observed behaviour); discuss and contribute to a preliminary HPSR framework for rehabilitation; identify emerging HPSR themes and preliminary research questions for an HPSR rehabilitation agenda; and identify enablers and barriers to building HPSR capacity in rehabilitation. The process of the meeting was informed by HPSR approaches to priority-setting [18].

### Participants

Potential participants were identified by members of the WHO Rehabilitation Programme through a pre-existing global database of rehabilitation stakeholders. Specifically, purposive sampling was employed to achieve maximum variation with regards to the regions, countries and types of organizations in attendance so that the consultation and final products would have relevance for the diverse needs of countries [19]. In addition, snowballing was employed to ensure that the list was comprehensive [19].

A summary of the types of stakeholders in attendance is provided in Table 1. There was representation from all WHO regions, with the highest representation from the WHO European Region. The majority of participants were from academia. Private sector and stakeholders with lived experiences of using rehabilitation were under-represented amongst the participants.

**Table 1:** Participant characteristics

Participant characteristics	Number of participants
Region excluding WHO staff (*N* = 95)	
Europe	29
The Americas	29
Western Pacific	18
Africa	10
South-East Asia	6
Eastern Mediterranean	3
Stakeholder type (*N* = 123)	
Academic institutions and journal editors	49
Government representatives	28
WHO staff	28
Rehabilitation professional organizations	14
Condition-specific organizations	3
Commercial entity	1

### Framing presentations

**Fig. 1:**
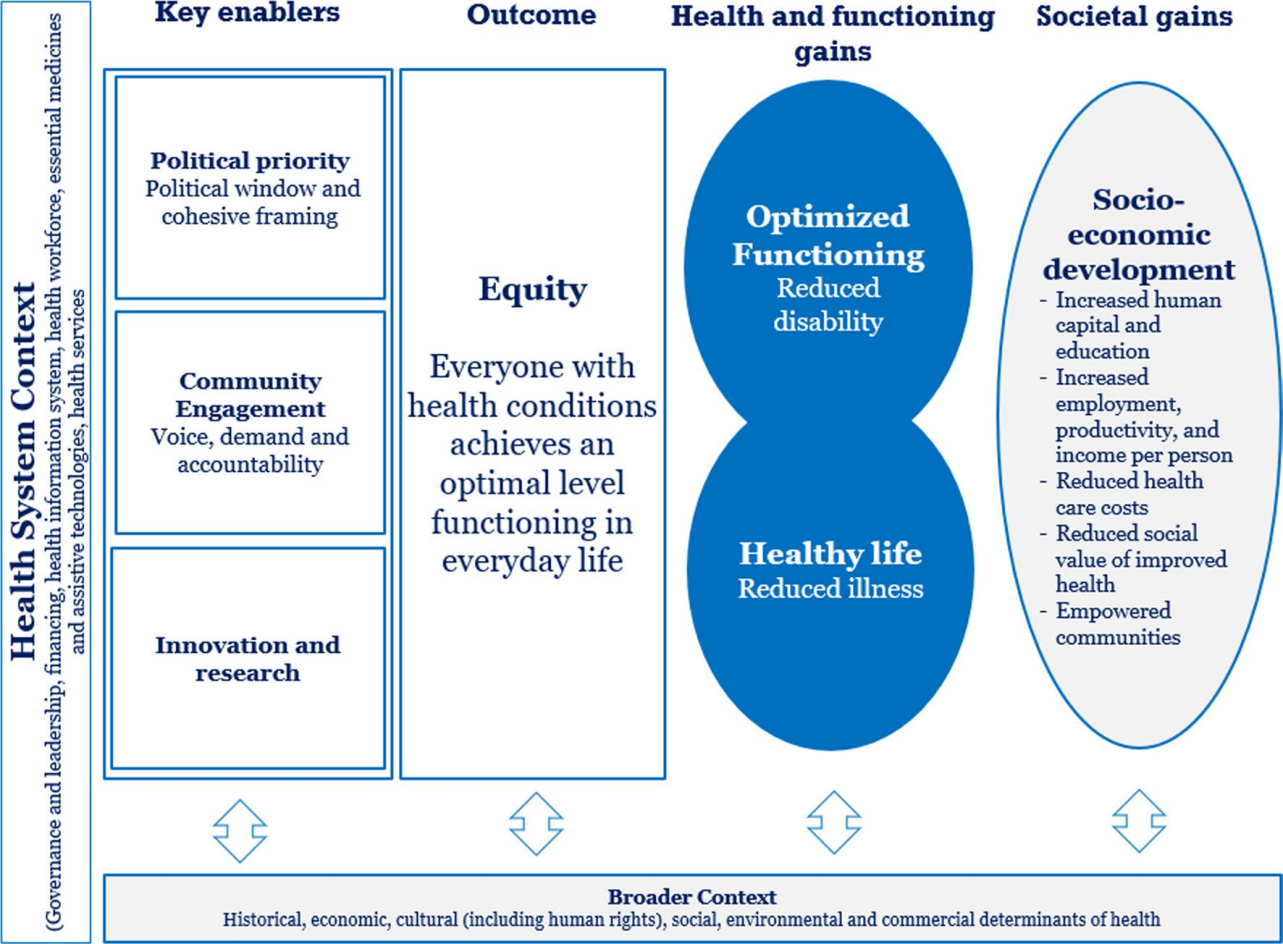
Preliminary HPSR framework presented to meeting participants for review. Source: Adapted from Stenberg et al. [20]

On day 1 of the meeting, participants were taken through a series of presentations to orient them to the imperative for developing an HPSR agenda for rehabilitation, systems-level design, evaluation and research questions, as well as potential enablers and barriers to advancing this HPSR agenda for rehabilitation. A preliminary HPSR framework for rehabilitation (Fig. 1) adapted from Stenberg et al. [20] was developed through an internal consultation and presented by the WHO Rehabilitation Programme to stakeholders as a means to stimulate thinking regarding further development of the framework. The preliminary framework identified political windows, cohesive framing, community engagement and innovation, and research as key enablers to integrating equitable rehabilitation into health systems. Additionally, both individual and societal benefits of equitable rehabilitation were highlighted as important components for consideration. These were acknowledged to be embedded within specific health system contexts, further embedded within the broader economic, social, environmental and political contexts.

### Technical consultations

On day 2, structured technical consultations took place. Participants were allocated to one of four multi-stakeholder groups with a facilitator to guide the structured technical consultations during which qualitative data in the form of written responses to guiding questions were collected. Participants first reviewed the preliminary research framework presented on day 1. Participants suggested changes to the framework to strengthen its relevance for rehabilitation. Groups then reconvened in the plenary and shared their findings. Next, and in groups, participants generated HPSR themes, research areas and appropriate system-level questions. Again, the groups reconvened in the plenary to share their findings.

### Technical working group

Following the research meeting, a technical working group was established consisting of members from the WHO Rehabilitation Programme, the Alliance, experts from the Johns Hopkins International Injury Research Unit, George Washington University, the University of Lucerne and the University of Geneva, to fully elaborate the HPSR rehabilitation framework. Ahead of the first meeting of the technical working group which took place over 2 days in Geneva during November 2019, data from the July 2019 meeting were systematically analysed. Thematic analysis [21] was conducted to identify emerging common themes regarding an HPSR agenda for rehabilitation, and related research areas. Feedback on the initial framework as provided by the participants was also reviewed and analysed.

The list of research questions identified during the structured technical consultations at the July 2019 meeting was also reviewed for its content. Research questions submitted by the participant groups were then organized to reflect similarities in the types of questions raised. This synthesis was presented to the technical working group, discussed and refined until a final list of preliminary research questions was agreed upon by all members. The technical working group continued to meet every 2 months in the succeeding 18 months.

## Results

### HPSR framework for strengthening rehabilitation

The resulting framework was informed by the common themes identified as being relevant for rehabilitation and their subsequent grouping into research areas. The research questions then demonstrate how the resulting framework may be used in practice.

Thematic analysis [21] of the data and information from the July 2019 meeting revealed the following common themes: (1) policy and governance; (2) political buy-in, including strengthening the Rehabilitation 2030 initiative; (3) equity and access; (4) research methodologies for rehabilitation; (5) resource allocation; (6) education, training and career pathways; (7) information systems for rehabilitation and measures for functioning; (8) integration and connection; (9) sustainability of rehabilitation services; and (10) family support and caregiver burden. Additionally, less common but important themes identified included UHC, primary care, human rights, and quality assurance in rehabilitation service delivery.

Research areas around these themes were synthesized and included (1) governance and leadership for rehabilitation through political buy-in and commitment, and embedding rehabilitation within the overall health and SDG agenda; (2) accessible, equitable and user-centred service delivery models that integrate rehabilitation within all levels of care, especially primary care; (3) availability of financial and nonfinancial resources to provide rehabilitation; (4) stakeholder engagement across various sectors, including health, finance, civil society, communities, end-users, public and private sectors; and (5) social support structures. The relationship between the common themes identified and the research areas is depicted in Table 2.

**Table 2:** Research areas depicted in relation to emerging common themes

Common themes	Research areas
Policy and governance Political buy-in, including strengthening the Rehabilitation 2030 initiative	Governance and leadership for rehabilitation through political buy-in and commitment, and embedding rehabilitation within the overall health and SDG agenda
Equity and access Research methodologies for rehabilitation	Accessible, equitable and user-centred service delivery models that integrate rehabilitation within all levels of care especially primary care
Resource allocation Education, training and career pathways Information systems for rehabilitation and measures/indicators for functioning	Availability of financial (insurance, subsidized cost) and nonfinancial (workforce, infrastructure, equipment and supplies, information systems) resources to provide rehabilitation
Integration and connection Sustainability of rehabilitation services	Stakeholder engagement across various sectors, including health, finance, civil society, communities, end-users, public and private sectors
Family support and caregiver burden	Social support structures (families and caregivers, employers, schools and neighbourhoods)

Based on this analysis, we present a framework to stimulate thinking on how HPSR approaches might increasingly support researchers, policy-makers and practitioners to address the pressing questions of how health systems can respond to the high need for rehabilitation in countries.

**Fig. 2:**
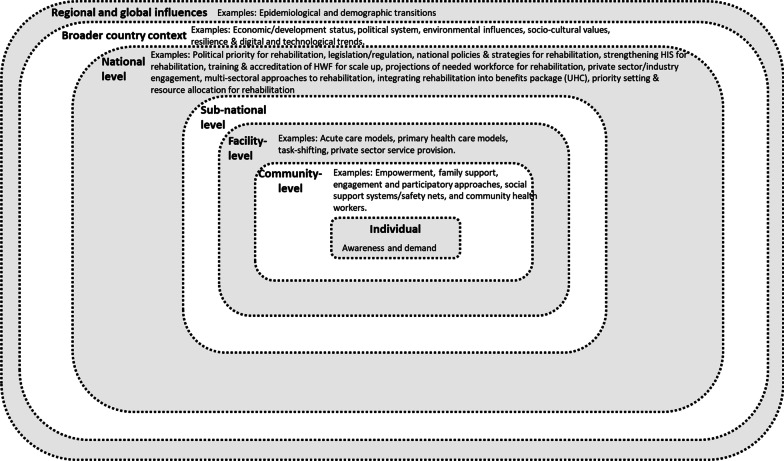
Proposed framework for stimulating HPSR in rehabilitation. What are the emerging research questions for HPSR approaches to rehabilitation?

The presented framework is informed by an ecological model, which recognizes the presence of multiple layers that may influence an individual [22]. Its (Fig. 2) multiple layers represent the embeddedness and continuity of health actions that connect rehabilitation at the level of the individual to broader interactions at community, facility, subnational, national and supranational levels. The individual level refers to users of rehabilitation. The community level expands the individual layer to include family units, communities and providers of care at the level of the community. The health facility level reflects the practices and stakeholders which directly or indirectly influence the provision of rehabilitation in a community’s health facilities. Depending on the organization of a country’s health system, the subnational level reflects administrative divisions, and their related health actors and actions, which encompass groups of health facilities and communities in which rehabilitation is provided. The national level takes a view of governmental actions and actors pertaining to the health sector which ultimately influence the rehabilitation accessed by individuals. The supranational level encompasses broader (i.e. beyond health) country, regional and global influences which influence the provision of rehabilitation.

While the primary focus of research might be at any level, it is important to note that these levels are not independent of one another. Rather, research findings in one level will undoubtedly have relevance for another. For example, findings which depict the national policy landscape of rehabilitation in a country will have implications for rehabilitation service delivery at the subnational and facility levels. Alternatively, there might be processes which occur at more than one level and thus require concurrent examination. An example of this might include assessing existing health facility assessment tools for rehabilitation services alongside a country’s overarching strategy for ongoing monitoring and evaluation of rehabilitation.

Since each of the levels exist within a broader context beyond the national level, research must account for these broader country, regional and global contexts which have a bearing on rehabilitation in a country. These considerations include political, social, economic, demographic and epidemiological factors. All these considerations of the levels and broader contexts might be represented in a framework as depicted in Fig. 2.

Given the above proposed framing, we outline several research questions for rehabilitation that are stimulated by HPSR approaches. The proposed research questions are presented alongside their related common themes and research areas (Table 3), although some questions relate to multiple research areas. We note that this is not an exhaustive list.

**Table 3:** Initial set of proposed questions to conduct rehabilitation HPSR presented with their related common themes and research areas

Common themes	Research areas	Proposed research questions
Policy and governance Political buy-in, including strengthening the Rehabilitation 2030 initiative	Governance and leadership for rehabilitation through political buy-in and commitment, and embedding rehabilitation within the overall health and SDG agenda	What is the political commitment for HPSR for rehabilitation at the national level to achieve SDGs and UHC? Where data is available, how do policy-makers and providers use evidence for implementation and modification of rehabilitation policies?
Equity and access Research methodologies for rehabilitation	Accessible, equitable and user-centred service delivery models that integrate rehabilitation within all levels of care, especially primary care	Are there national or subnational standards for rehabilitation care? What are the current resources for funding HPSR for rehabilitation at various levels of care? What additional resources are needed to conduct HPSR for rehabilitation? What is the level of preparedness of healthcare facilities to implement rehabilitation care-related policies and procedures? How is technology used to provide rehabilitation care?
Resource allocation Education, training and career pathways Information systems for rehabilitation and measures/indicators for functioning	Availability of financial (insurance, subsidized cost) and nonfinancial (workforce, infrastructure, equipment and supplies, information systems) resources to provide rehabilitation	How can the health workforce be organized to scale up rehabilitation? What are the current policies and gaps in evidence translation related to HPSR for rehabilitation? What are the current accreditation requirements for rehabilitation providers? To what extent are assistive technologies available in the provision of rehabilitation? What is needed to strengthen health information systems (HIS) to include data on rehabilitation?
Integration and connection Sustainability of rehabilitation services	Stakeholder engagement across various sectors, including health, finance, civil society, communities, end-users, public and private sectors	Which policies exist to ensure equity in rehabilitation, and how does their implementation affect access (multilevel analysis)?
Family support and caregiver burden	Social support structures (families and caregivers, employers, schools and neighbourhoods)	What are the enablers and barriers related to access, coverage, and quality of rehabilitation care?

Inherent to HPSR is its question-driven nature. This means that the methods to be used will serve the questions being asked, and not vice versa. Therefore, in answering these questions, there is a multiplicity of research methods and approaches that can be used, including quantitative, qualitative and mixed methods [12, 23].

## Discussion

This paper reports on the outcome of the global consultative meeting on HPSR for rehabilitation which took place following the second Global Rehabilitation 2030 meeting. In particular, this paper proposes a framework to stimulate HPSR in rehabilitation with the intention to align the discourse regarding rehabilitation with a more systemic perspective. This supports current evidence which examines rehabilitation from a health systems perspective [24], and goes further to guide how countries might begin to conduct such a systemic inquiry.

The framework and research questions support the goals of the Rehabilitation 2030 agenda by facilitating evidence-informed policy implementation, which in turn supports policy-makers’ efforts to strengthen health systems for rehabilitation [11, 25]. The demand for HPSR evidence amongst policy-makers is increasing [26], and as such, the need for systemic evidence for and approaches to rehabilitation has never been greater.

The presented framework lends itself for use in its current or an adapted form by a range of stakeholders. First, policy-makers may identify where additional research is needed to inform rehabilitation-relevant policy development and implementation. Second, researchers in collaboration with policy-makers may use this framework to guide areas for research inquiry relevant to rehabilitation in countries. Third, rehabilitation professional organizations can generate hypotheses about the forces that are important for training and deploying a rehabilitation workforce. Fourth, civil society might employ the framework to identify what advocacy areas need special attention when addressing equitable access to high-quality rehabilitation in countries.

Additional considerations ought to be addressed when discussing an HPSR framework for rehabilitation. The first consideration relates to whether and how equity is promoted through the exercise [27]. Pratt et al. [28] argue that global health research informs what interventions health systems can provide, which then inform what interventions are made available to the population. Within the context of rehabilitation, this means that research should prioritize those with the highest unmet needs in order to optimize their functioning and thus improve their overall health. Therefore, HPSR in rehabilitation can usefully address the reduction of existing inequities related to accessing rehabilitation worldwide. Relatedly, this work may encourage a similar shift in funding for rehabilitation research towards systems research.

Another consideration that ought to be addressed is the process for stakeholder engagement and the extent to which decision-making is truly shared. The framework development process ensured that it captured contextually relevant information related to countries and was up to date with the current discourse on HPSR. Thus, a combination of bottom-up and top-down approaches were used in its development. This iterative process included top-down approaches to synthesize the relevant literature to present a preliminary framework. This was then followed by a bottom-up process for the generation of themes and questions from a diverse set of stakeholders. Finally, a top-down approach enabled the synthesis of all the collected themes and questions. The process therefore exemplified shared decision-making in the development of the framework [28]. In practice, since the framework is universal, it is applicable to all settings regardless of income status. It will be within the remit of countries themselves to define specific research questions from this framework which apply to their own populations’ health profiles, health system organization, funding arrangements, and requirements for achieving equity targets within their own contexts.

The proposed framework for stimulating HPSR in rehabilitation has several strengths. First, the participatory approach involving a diverse set of stakeholders from several countries and professional contexts ensured that the resulting framework and questions were relevant to all countries’ health systems. Second, the framework goes beyond the six WHO traditional building blocks of the health system (service delivery, health workforce, health information systems, access to essential medicines, financing and leadership/governance) [29] to include multiple sectors. Third, it allows for users to identify the diversity of outcomes and their complexity at multiple levels. The proposed framework also has limitations. First, it is not exhaustive. Therefore, additional aspects not covered in it are likely to be identified during the research process in countries. Second, it does not explicitly address the relational aspects between health systems actors. However, since all aspects of health actions are ultimately contingent on people, this means that research should also aim to describe these relationships between health systems actors and their impacts on rehabilitation in countries. Third, the framework does not explicitly address strengthening capacity for conducting HPSR for rehabilitation. Even so, WHO is addressing this through several initiatives, including a forthcoming special issue on HPSR for rehabilitation in the WHO Bulletin [30], and by promoting HPSR in countries where WHO provides technical support to strengthen rehabilitation. As an outcome of these efforts, the United States Agency for International Development (USAID) launched a call for proposals for HPSR in rehabilitation [31]. As a result, the project Learning, Acting and Building for Rehabilitation in Health Systems (ReLAB-HS), a 5-year, US$ 39.5 million, multicountry study initiated in 2021, has a focus on HPSR capacity-strengthening for rehabilitation [32].

## Conclusion

In conclusion, we reported on the outcome of a global consultative process which aimed to advance HPSR for rehabilitation. Through a participatory multi-stakeholder approach, a framework was proposed to stimulate HPSR in rehabilitation in countries in order to generate policy-relevant evidence. Additionally, an initial set of questions which demonstrate how the framework might be used in countries was proposed. We anticipate that this work will make a critical contribution towards building HPSR capacity and generating evidence for rehabilitation by shifting the narrative of rehabilitation towards more systemic approaches in health systems.

## Data Availability

The datasets used and/or analysed during the current study are available from the corresponding author on reasonable request.
